# Decision Models and Technology Can Help Psychiatry Develop Biomarkers

**DOI:** 10.3389/fpsyt.2021.706655

**Published:** 2021-09-09

**Authors:** Daniel S. Barron, Justin T. Baker, Kristin S. Budde, Danilo Bzdok, Simon B. Eickhoff, Karl J. Friston, Peter T. Fox, Paul Geha, Stephen Heisig, Avram Holmes, Jukka-Pekka Onnela, Albert Powers, David Silbersweig, John H. Krystal

**Affiliations:** ^1^Department of Psychiatry, Harvard Medical School, Brigham and Women's Hospital, Boston, MA, United States; ^2^Department of Anesthesiology and Pain Medicine, Harvard Medical School, Brigham and Women's Hospital, Boston, MA, United States; ^3^Department of Psychiatry, Yale University, New Haven, CT, United States; ^4^Department of Anesthesiology and Pain Medicine, University of Washington, Seattle, WA, United States; ^5^Department of Psychiatry, Harvard Medical School, McLean Hospital, Belmont, MA, United States; ^6^Department of Psychiatry, University of Washington, Seattle, WA, United States; ^7^Department of Biomedical Engineering, Faculty of Medicine, McConnell Brain Imaging Centre (BIC), Montreal Neurological Institute (MNI), McGill University, Montreal, QC, Canada; ^8^Mila—Quebec Artificial Intelligence Institute, Montreal, QC, Canada; ^9^Medical Faculty, Institute of Systems Neuroscience, Heinrich Heine University Düsseldorf, Düsseldorf, Germany; ^10^The Wellcome Centre for Human Neuroimaging, Institute of Neurology, University College London, London, United Kingdom; ^11^Research Imaging Institute, University of Texas Health, San Antonio, TX, United States; ^12^Departments of Psychiatry, University of Rochester Medical Center, Rochester, NY, United States; ^13^T.J. Watson IBM Research Laboratory, Yorktown Heights, NY, United States; ^14^Department of Neurology, Icahn School of Medicine, New York, NY, United States; ^15^Department of Psychology, Yale University, New Haven, CT, United States; ^16^Department of Biostatistics, T. H. Chan School of Public Health, Harvard University, Boston, MA, United States

**Keywords:** psychiatry, biomarker, digital phenotype, diagnosis, Bayesian inference, decision model

## Abstract

Why is psychiatry unable to define clinically useful biomarkers? We explore this question from the vantage of data and decision science and consider biomarkers as a form of phenotypic data that resolves a well-defined clinical decision. We introduce a framework that systematizes different forms of phenotypic data and further introduce the concept of decision model to describe the strategies a clinician uses to seek out, combine, and act on clinical data. Though many medical specialties rely on quantitative clinical data and operationalized decision models, we observe that, in psychiatry, clinical data are gathered and used in idiosyncratic decision models that exist solely in the clinician's mind and therefore are outside empirical evaluation. This, we argue, is a fundamental reason why psychiatry is unable to define clinically useful biomarkers: because psychiatry does not currently quantify clinical data, decision models cannot be operationalized and, in the absence of an operationalized decision model, it is impossible to define how a biomarker might be of use. Here, psychiatry might benefit from digital technologies that have recently emerged specifically to quantify clinically relevant facets of human behavior. We propose that digital tools might help psychiatry in two ways: first, by quantifying data already present in the standard clinical interaction and by allowing decision models to be operationalized and evaluated; second, by testing whether new forms of data might have value within an operationalized decision model. We reference successes from other medical specialties to illustrate how quantitative data and operationalized decision models improve patient care.

Biomarkers are crucial to medical science, so much so that even the U.S. Congress has sought to define them. The National Institutes of Health (NIH) defines a biomarker as “a characteristic that is objectively measured and evaluated as an indicator of normal biologic processes, pathogenic processes, or pharmacologic responses to a therapeutic intervention” (1). The U.S. Congress and Food and Drug Administration (FDA) further defined a biomarker as “a physiologic, pathologic, or anatomic characteristic or measurement” that “includes a surrogate endpoint” (2) that indirectly reflects a primary disease process. So defined, identifying and applying a biomarker in clinical practice requires that a bottom-up knowledge of pathophysiology converge and meaningfully interact with a clinician's top-down evaluation of phenomenology. A biomarker, therefore, presupposes that pathophysiology interact with phenomenology, thereby allowing clinicians to apply physiologic tools to the diagnosis and treatment of a patient's disease.

Though the American Psychiatric Association has regularly published consensus reports outlining promising biomarkers (3), the way clinicians diagnose and treat psychiatric disease remains largely unchanged. Outside of neurodegenerative conditions, no psychiatric disorder requires or has available a quantitative biomarker to establish a diagnosis, stage the progression of illness, guide the selection of treatment, or evaluate the impact of treatment (65).

Here, we suggest that the failure to define useful biomarkers rests in part on diagnostic procedures that, in their current form, cannot be fully operationalized. In turn, we argue that psychiatry's inability to operationalize clinical decision results from a reliance on imprecise, qualitative data and on data-gathering procedures that are unique to each clinician. Though this failure further suggests the need for advances in our bottom-up understanding of pathophysiologic mechanisms, here, we focus primarily on improving the clinician's top-down evaluation and diagnosis. To explicate this view, we define a series of basic concepts and build upon these concepts to show why biomarkers remain elusive in psychiatry and how we might proceed.

## Phenotypes and Decision Models, Defined

**Figure 1:**
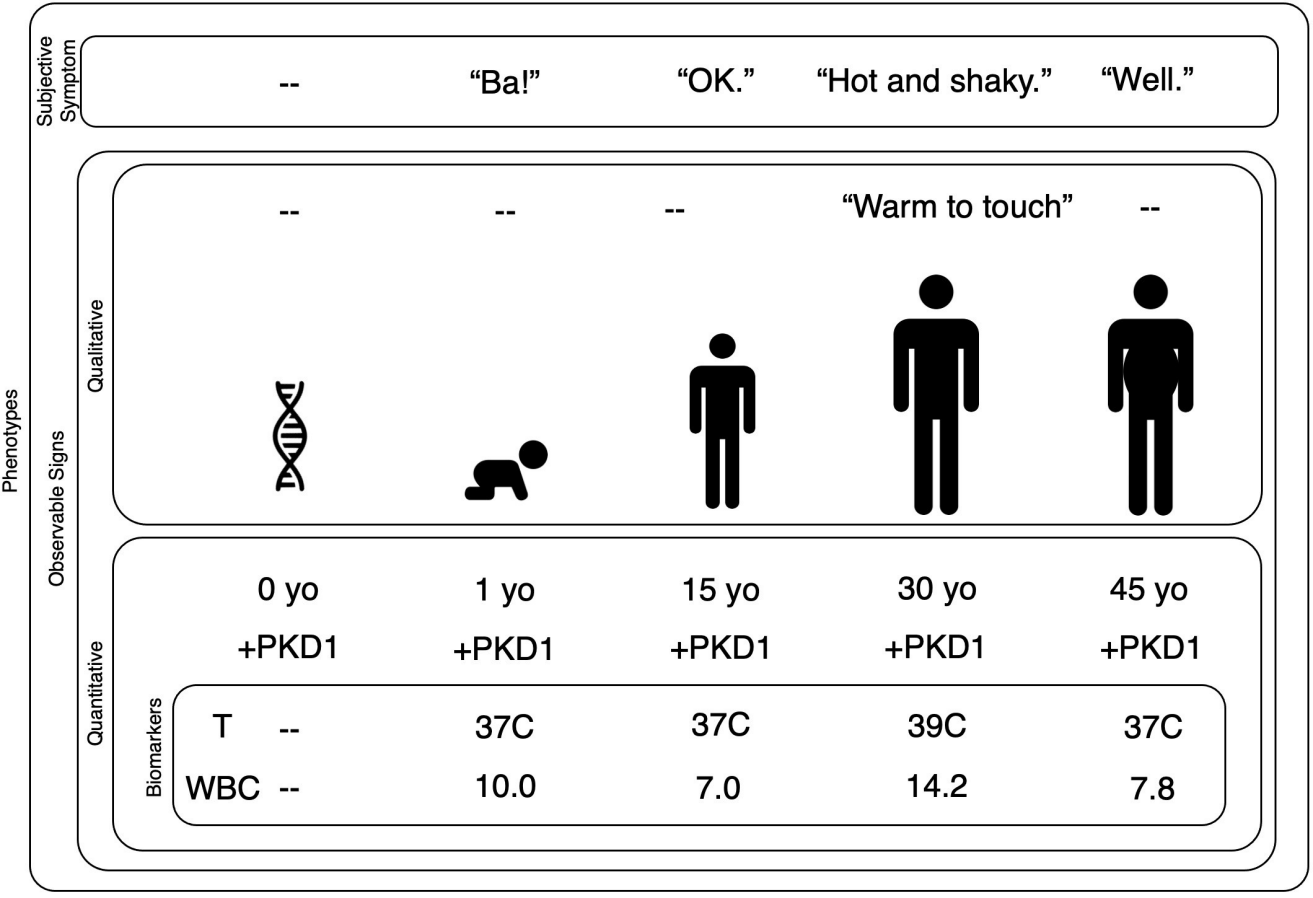
Biomarkers are quantified phenotypes relevant to a decision model. This nested plot shows that subjective symptoms and observable signs are phenotypes. Signs can be observed with qualitative or quantitative methods. A quantitative observable sign requires the use of an instrument to measure the data of interest. A biomarker is a quantitative observable sign that has some bearing on a given decision model. In this case, we are trying to evaluate a 30-year-old man who reports he is “hot and shaky.” A clinician might observe his habitus and record that the man's skin is “warm to touch.” Quantitative observable signs might include his age and the presence of the PKD1 gene. Our clinical goal is to understand and treat his report of “hot and shaky,” therefore, within our decision model, his age and PKD1 gene status are not necessarily relevant. His temperature and WBC are relevant because they have direct bearing on our decision model. Note that a phenotype can change over time as one's genes interact with the environment: the symptoms and signs of a bacterial infection are phenotypes that emerge only during the illness. Further note that while some phenotypes may change year-to-year or even moment-by-moment, the autosomal dominant mutation at the PKD1 gene on chromosome 16 will not change. PDK, Polycystic Kidney Disease; yo, year-old; T, temperature; C, Celsius; WBC, White Blood Count (reported as ×10^3^/μL).

Broadly speaking, a phenotype encompasses any observable characteristic, from an individual's molecular and biochemical properties to their repertoire of possible behaviors (4). In psychiatry, clinically relevant phenotypes are generally conceptualized as symptoms and signs (see Figure 1) (5).

Symptoms are reported by the patient (e.g., “I feel hot.”) and rely on a patient's ability to sense, interpret, and convey their personal experience. Conversely, signs can be qualitatively or quantitatively observed, e.g., skin that is qualitatively “warm to the touch” can be quantified as 39°C. In the case of a qualitative sign, the sensor is the clinician's eyes, ears, or fingers; the clinician senses and summarizes the data at hand by noting that the skin is “warm to the touch.” In the case of a quantitative sign, the sensor is an instrument designed to measure the phenomenon of interest; e.g., a thermometer records that the skin is 39°C.

A biomarker is a quantitative sign that, as stated above, captures some aspect of biology that is salient to health or disease. Broadly speaking, there are two classes of biomarkers: descriptive and treatment. Descriptive biomarkers screen for disease or stage disease progression (see Table 1). Treatment biomarkers inform therapeutic interventions that, based on their relationship to pathophysiology can be palliative, modifying, or curative. Because a biomarker's overall goal is to inform clinical reasoning, to the NIH's definition we add that a biomarker must help resolve a well-defined clinical decision within what we will call a “decision model” (33).

**Table 1:** Types of biomarkers.

**Class**	**Purpose**	**Goal**	**Example**
Descriptive	Screening	Indicate a possible disease process	Fever → motivates further workup
	Staging	Indicate disease stage (without explicitly informing treatment)	Creatinine → kidney disease progression
Therapeutic	Palliative	Inform treatment that does not act on pathophysiology	Painful metastatic cancer → morphine
	Modifying	Inform treatment that modifies pathophysiology	Hypertension → Anti-hypertensive
	Curative	Inform treatment that cures pathophysiology	HER-2 positivity → Herceptin MRSA → Vancomycin

We introduce the term “decision model” to describe the series of strategies and policies that a clinician uses to evaluate a patient and craft a treatment plan (33). These strategies and policies can be acquired explicitly through instruction (whether clinical training or review of scientific literature) or implicitly through clinical experience. As the term suggests, a decision model informs a clinician's decision about how to seek out, combine, and act on clinical data. Within a decision model, phenotypic data inform hypotheses of how those data interrelate and guide the clinician's thoughts and actions during the exam, the purpose of which is to decide how best to intervene with treatment (6). Therefore, a decision model is fluid, evolving continuously as new data become available.

Clinical data can be assessed based on their reliability and utility; put differently, data are not equally reliable or useful. Symptoms are subjective, being a patient's expression of their personal experience. Signs are objective, being observed either by a clinician or by an instrument designed for that purpose (7). The reliability of a symptom or a sign depends on how accurately it captures a given phenomenon; in the case of a patient, how faithfully he reports his personal experience; in the case of a clinician, how skillfully she senses “warm to the touch” and a thermometer's calibration to degrees Celsius. To be of value within a decision model, data must be reliable and useful. A clinician might observe that a patient has freckles however this datapoint is unlikely to be useful in a decision model for schizophrenia; the number of freckles, therefore is unlikely to serve as a useful biomarker for schizophrenia staging or treatment. How reliably a biomarker answers a clinical question can be further assessed in terms of sensitivity, specificity, and accuracy. What makes a biomarker clinically valuable will be further discussed in a separate section, below, however, it is worth noting that once a biomarker has met acceptable criteria for reliability, it might transition to a standard clinical test that in addition relies on the accuracy, range of error, and uncertainty of the assay, instrument or clinical tool.

Decision models can be assessed based on their efficacy and efficiency. An effective decision model will improve a patient's clinical state. Because clinical work is temporal in nature (i.e., ineffectively treated disease states can progress and worsen), efficiency is an important value for a decision model. The efficiency of a decision model can be assessed by how much time and data gathering are required to reach an optimal decision. Assuming that two decision models are equally effective, a decision model that requires 5 min to gather 10 datapoints is more efficient than one that requires 20 min to gather 100. The importance of these criteria will become clearer, below, as we discuss formalizing and optimizing decision models.

The interaction of subjective symptoms, observable signs, and biomarkers within a decision model can be illustrated with a simple example: a patient presents to an emergency room reporting “I have the worst headache of my life.”

The patient's symptom report serves as the first datapoint in the clinician's decision model for evaluating and treating the patient's headache. The clinician will populate her decision model with hypothetical causes of experiencing the “worst headache of my life” (e.g., subarachnoid hemorrhage, migraine, infection) and will use her decision model to systematically eliminate or confirm hypotheses by selectively soliciting other symptoms and signs. Data from the patient about when the headache began and whether they've had similar headaches or recent head trauma will no doubt be paired with data observed by the clinician looking for focal neurologic deficits or measurements of temperature, blood pressure, electrolyte, and other laboratory panels. Together, these data will guide the clinician's headache decision model.

Viewed from a Bayesian perspective (8, 9), a clinician begins with a prior belief or an a-priori probability that a given disease best explains the available data based on the patient's report, clinical appearance, or disease prevalence in that clinical setting. By selectively searching for and building in additional data to her overall decision model, the clinician continuously updates the likelihood that these data can be best explained by any specific disease. As she updates these likelihoods, she decreases her uncertainty about how to treat the patient.

Technically speaking, this is a process of Bayesian belief updating that underwrites most forms of data assimilation and uncertainty quantification in the life and physical sciences—sometimes referred to as evidence accumulation (10–14). As we will see below, this process of belief updating can be cast in terms of converting a prior belief (before seeing any clinical data) into a posterior belief (after seeing the data), in a principled fashion [for a more technical example of Bayesian statistics, see (15)].

Data do not have equal utility within a decision model. In an emergency room patient who reports “worst headache of my life,” the presence of fever is causally non-specific. Put differently, fever is weakly specific for multiple causes of disease. Fever might prompt a clinician to collect additional types of data, such as an analysis of cerebrospinal fluid or blood. These data are also weakly specific for a given disease cause, but as weakly specific data accumulate, the additive effect is to increase the overall likelihood of one hypothesis over competing hypotheses. For example, if a cerebrospinal fluid analysis show high levels of glucose, white blood cells and protein, these data suggest that the person's headache is caused by a bacterial meningitis. Should a cerebrospinal fluid culture identify a specific type of bacterial infection, a clinician might treat this condition with an antibiotic drug that has known efficacy against that bacteria. In this example, the clinician combined multiple forms of weakly specific data within her decision model to converge on an appropriate treatment. The process of selectively combining weakly specific though complimentary datapoints and of moving from subjective symptom to observable signs to treatment is at the heart of the medical enterprise.

Technically, this process is beautifully described in terms of the principles of optimal Bayesian design (16); namely, the clinician gathers data that she believes will most efficiently resolve her uncertainty about competing hypotheses and, overall, about how to act to treat her patient. In machine learning, this is known as the problem of active learning; namely, finding the next data point that is maximally informative in relation to beliefs about how the data were caused (12). In the neurosciences, this is known as active inference; namely, responding to epistemic affordances offered by different diagnostic avenues (17). The key problem addressed by these approaches to diagnosis is that the best data to solicit is determined by the beliefs or hypotheses currently entertained by the clinician, which is to say, by the clinician's current decision model. In other words, only if the clinician must decide whether a bacterial meningitis might have caused her patient's specific phenotype (comprising: “headache,” fever, etc.), will she order a cerebrospinal fluid culture to test this hypothesis. A cerebrospinal fluid culture is not the indicated diagnostic procedure across decision models, but it is a *useful* test based on the data that the clinician has already assimilated.

In short, data are not of equal utility to all decisions within a larger decision model. The presence of a fever might be relevant to prompt further workup, but not immediately relevant to antibiotic selection. Data have utility only within the context of a specific clinical decision (18, 19). Only in rare cases do single datapoints or single forms of data independently resolve clinical decisions.

On this view, biomarkers have a special (epistemic) value because they resolve uncertainty under a particular decision model. The value of a biomarker is not in identifying a disease in isolation from other clinical data; but rather, a biomarker operates within and updates an existing decision model and, therefore, collaborates with other clinical data to decrease the overall uncertainty of a well-defined course of action.

## Candidate Biomarkers: Associative and Predictive

We broadly consider associative and predictive biomarker studies and we evaluate whether and how they could operate within decision models in psychiatry (65).

Associative biomarker studies rely on classic null-hypothesis tests to compare group means of a given parameter and, therefore, associate that parameter with a disease group. An example is whether brain structure in a group of depressed patients differs from a group of non-depressed controls (20) or whether genetic variants of the serotonin transporter gene differ in depressed and non-depressed patients who have experienced life stressors (21, 22). Other work has attempted to trace the emergence of depression by collating independently collected genetic, cellular, and whole-brain imaging datasets (23). So far, associative biomarkers have offered little clinical utility in psychiatry; the statistical methods upon which they are based are formulated at the group or population rather than the individual level. Associative biomarkers can be actionable on the individual level, but they must first be evaluated in new individuals and separate cohorts as a predictive biomarker. One example is the North American Prodromal Longitudinal Study risk calculator, which associated phenotypic variables (e.g., cognitive deficits and symptom profiles) with the risk of transitioning from clinical high risk to psychosis (24); this study is currently being evaluated in new individuals and separate cohorts as a predictive biomarker.

Predictive biomarker studies use specialized methods to identify values (whether quantitative or subjective) within a dataset, which, in combination, predict a desired variable such as a diagnosis or clinical outcome (25). For example, machine-learning models trained on a large group can be validated and applied to individuals (26). A supervised machine-learning model sieves through many candidate variables to identify which are most predictive of a disease-related target variable. An example is a recent supervised machine-learning study that identified a pattern of life experiences, neurobiological differences, and personality traits that were predictive of binge drinking in 14-year-olds (27). An unsupervised machine-learning model looks across a similarly large number of candidate features to identify patterns that can then be assessed for common properties. Unsupervised machine-learning models are said to be unbiased and data-driven because they do not require data to be labeled *a priori* or a user to specify an outcome of interest. For example, a recent unsupervised machine-learning study identified three co-occurring symptom clusters across patient self-report and clinician-rated symptom scales that were associated with response to antidepressant and/or cognitive behavioral therapy (28, 29).

There is notable variability across the features and target variables currently explored in psychiatric biomarker development: subjective symptoms (patient self-report or behavioral trait scales) are often paired with quantitative observable signs (brain imaging, genetics, age). These features and target variables, in turn, are often evaluated within the context of a psychiatric diagnosis (see Box 1), which is largely based on subjective symptoms. The variability in features and target variables, therefore, could in part be explained by the field trying to define an unknown clinical landscape: because it is unclear how to best conceptualize psychiatric disorders, it is further unclear which data might afford the greatest utility in understanding them.

Box 1 **Diagnostic Foraging.**

*Attempts to classify psychiatric disease have primarily focused on subjective symptoms and qualitative, observable signs. The Diagnostic Statistical Manual and International Classification of Disease use expert consensus to classify mental illnesses into binary disease categories based on combinations of subjective symptoms and observable signs* (30, 31). *Biomarker development has no doubt been stymied by an unavoidable corollary of combinatorial diagnostic groups: the sheer number of possible symptom combinations meeting criteria. For example, a recent commentary on the ethical implications of machine learning in psychiatry computed that there are 7,696,580,419,045 unique sets of symptoms that meet criteria for schizophrenia as defined in the Structured Clinical Interview for DSM-5 (SCID-5)* (32). *Similarly, because there are at least 488,425 ways to be diagnosed with a major depressive episode based on DSM-4, such top-down phenotypic imprecision was likely a reason that the first treatment-selection biomarker trial did not succeed* (33). *Though top-down combinations of symptoms have, in other disciplines, converged with bottom-up pathophysiology (e.g., the pill-rolling tremor, masked facies, festinating gait, and stooped posture that are pathognomonic of Parkinson's Disease and substantia nigra degeneration) sometimes they have not (e.g., dropsy). Practically, it would appear difficult to bridge bottom-up pathophysiology and seven trillion symptomatically dissimilar schizophrenias*.

*Other strategies suggest that behavior might be more accurately captured by considering multiple dimensions of a disease (e.g., mood state)* (34) *along a continuum. Yet other studies suggest that the very act of diagnosing is poorly framed and that an individual's symptom profile might be better captured with a single dimension, such as “p”* (35). *The Hierarchical Taxonomy of Psychopathology is an attempt to quantitatively define constellations of co-occurring signs, symptoms, and maladaptive traits and behaviors that might prove useful to clinical assessment and treatment* (6, 36).

*None of these taxonomies references a quantitative biomarker or attempts to define a quantitative threshold for separating a disease state from a non-disease state. As in the case of blood pressure, as the field moves toward greater understanding such a threshold will likely change, however, in the absence of quantitative measures, such a threshold cannot be evaluated and refined*.

Notwithstanding the wide range of data across studies, the data evaluated in individual biomarker studies is narrow. Most studies associate a single form of data (e.g., genetic or neuroimaging or symptom assessment) with diagnosis. Even complex machine-learning studies that combine multiple forms of data are relatively narrow compared to the range of data a clinician routinely gathers. Although machine learning studies may provide novel insights into mental illness, they often fail to replicate and, thus far, have failed to guide clinical practice.

This is not surprising; single datapoints or even single forms of data rarely resolve a clinical course of action, even when the range of possible courses of action is known and well-described (e.g., because the common types of infection and treatment are known, there was a much smaller number of possible clinical decisions in our “hot and shaky” patient than for a given psychiatric patient, wherein the landscape is not known). Furthermore, there is a growing appreciation of the limitations of machine learning in terms of “explainability” and difficulties establishing the predictive validity of a simple set of biomarkers. With the exception of machine learning procedures based upon generative models (e.g., variational auto encoders or generative adversarial networks), most schemes suffer from the poor generalization, predictive validity, and overfitting that go hand-in-hand with an overly parameterized deep learning network.

To put it more plainly, associative biomarker studies suffer from the fallacy of classical inference, wherein an overpowered group identifies a candidate biomarker with a high statistical significance but with an effect size that is very small and essentially disappears at the single subject level. Meanwhile, predictive biomarker studies can overfit the parameters of their model to a given dataset; therefore, even though a predictive biomarker might explain a large amount of the variance, this model is useless in a novel clinical population. Although cross-validation techniques are meant to help minimize the likelihood of overfitting (25, 37), many datasets are unique, so cross-validating on an independent but similarly unique dataset does not truly demonstrate generalizability or resolve the overfitting problem (38). Grounding biomarker studies in clinical practice and making utility within a decision model a necessary component of biomarker development, therefore, might prove helpful. These technical considerations bring us back to the question of value: what gives a biomarker value and which data offer the most value to a decision model?

## What Gives a Biomarker Clinical Value?

Biomarkers have value if they help clinicians better describe or better treat disease within a larger decision model (see Table 1). Unfortunately, many candidate biomarkers attempt to describe disease solely in terms of diagnosis, a pursuit that has been complicated by a lack of consensus about the best way to diagnose psychiatric diseases (see Box 1). Indeed, it is an understandably complex (if not impossible) task to develop a biomarker for schizophrenia when there are over 7.6 trillion unique combinations of symptoms that each meets diagnostic criteria for schizophrenia (32).

For a biomarker to have value, it should guide clinical decision independent of diagnosis. In medicine, descriptive biomarkers can help screen for or stage a disease. Meanwhile, therapeutic biomarkers can guide clinical decision toward palliative, modifying, or curative treatments.

Palliative therapeutic biomarkers identify treatments that suppress the downstream manifestations of a disease: for example, an opioid might be prescribed for pain related to metastases from a HER-2 positive cancer. Palliative therapeutic biomarkers are broadly applicable across diseases because they are not related to any specific pathophysiology; opioids relieve pain related to many pathophysiologies and so a biomarker indicating that an opioid is an appropriate clinical course of action would be applicable to many diseases. Because they do not treat but rather suppress the expression of pathophysiology, many current psychiatric treatments fall in this category. For example, hydroxyzine might suppress the panic of someone with generalized anxiety disorder, but it is unlikely that panic is related to dysregulation of the histaminergic system. Or furthermore, antipsychotics and antidepressants are broadly used across psychiatric diseases because they most likely suppress the downstream effects of (rather than modify) pathophysiology. Fortunately for our patients, the majority of psychiatric therapies require little or no understanding of pathophysiology because they target downstream mechanisms that are found broadly across disorders.

Modifying and curative therapeutic biomarkers identify subsets of patients that share a pathophysiology, allowing them to be paired with treatments that target that pathophysiology. While modifying treatments temporarily (dependent on the duration of action), a curative treatment eliminates or reverses the pathophysiology. Such therapeutic biomarkers apply to a progressively narrower patient population because they would identify, in essence, a subset of a larger population that, in the absence of a biomarker, would appear clinically similar. For example, blood pressure is a valuable biomarker because without it, a clinician might not know to prescribe an otherwise well-appearing patient an anti-hypertensive.

The more deep our knowledge of bottom-up pathophysiology, the more specific the possible treatment and the less likely the associated biomarker is to be broadly applicable to the larger population. Put differently, the rarer a given pathophysiology is, the less likely a therapeutic biomarker is to provide actionable insights to the vast majority of patients. For example, research suggests that within the larger category of schizophrenia, there are the very rare Mendellian risk genes (e.g., 22q11 or GRIN2A) and the relatively more common (though still quite rare) polygenic common risk loci (39). Should treatments be identified for each specific pathophysiology, it seems unlikely that they would be applicable to the larger population of “schizophrenia,” for which there are ~7.6 trillion possible combinations of symptoms and signs (32). Likewise, testing any biomarker for a specific schizophrenia pathophysiology on a sample drawn from ~7.6 trillion possible schizophrenias lacks face validity and is unlikely to yield positive or reproducible conclusions. Such prima facia logic suggests the need for greater phenotypic precision within operationalized decision models; in other words, for more serious consideration of the clinical data and how these data are integrated to articulate specific decisions.

Overall, the need for top-down biomarkers will grow in importance as our knowledge of bottom-up pathophysiology advances. In other words, as we deepen our understanding of the complex pathophysiology underlying phenomena of psychiatric disease, we anticipate a series of treatments that modify or cure a mechanistically precise pathophysiology. Identifying patients who could benefit from such modifying or curative treatments will require biomarkers that operate within clinical decision models. A primary task facing psychiatry, therefore, is determining which data offer the most value to a decision model.

Consider that a standard psychiatric interview gathers information about a patient's biologic, psychologic, and social history (40). A clinical evaluation might yield thousands of heterogenous datapoints that can range from: a patient's observable behavior; their reported narrative and symptomatology; results from clinical tests like blood work, urine toxicology, electrocardiogram; reports from family members, legal authorities, or other healthcare providers; the patient's socioeconomic status; and how these data change over time. Any of these data might have utility within a decision model, depending on the clinical setting and the clinician's training and experience.

Sifting through clinical data to operationalize decision models that can be tested and optimized has always been a fundamental complexity of medicine. Historically, successful strategies to develop decision models and biomarkers have been firmly rooted in physiology or in epidemiology.

## Biomarker Development Strategies: Physiology and Epidemiology

A biomarker bridges bottom-up pathophysiology and top-down phenomenology. Two strategies to biomarker development have been based in physiology and epidemiology (65). The physiology-based strategy can be considered a bottom-up approach wherein understanding the pathophysiology of a well-defined decision model leads to an understanding of how to clinically intervene. The epidemiology-based strategy can be considered a top-down approach where, in the absence of a well-defined decision model, identifying common phenomena that precede a defined clinical outcome leads to a better understanding of disease and, therefore, to identifying useful therapeutic targets. We explore both below.

**Figure 2:**
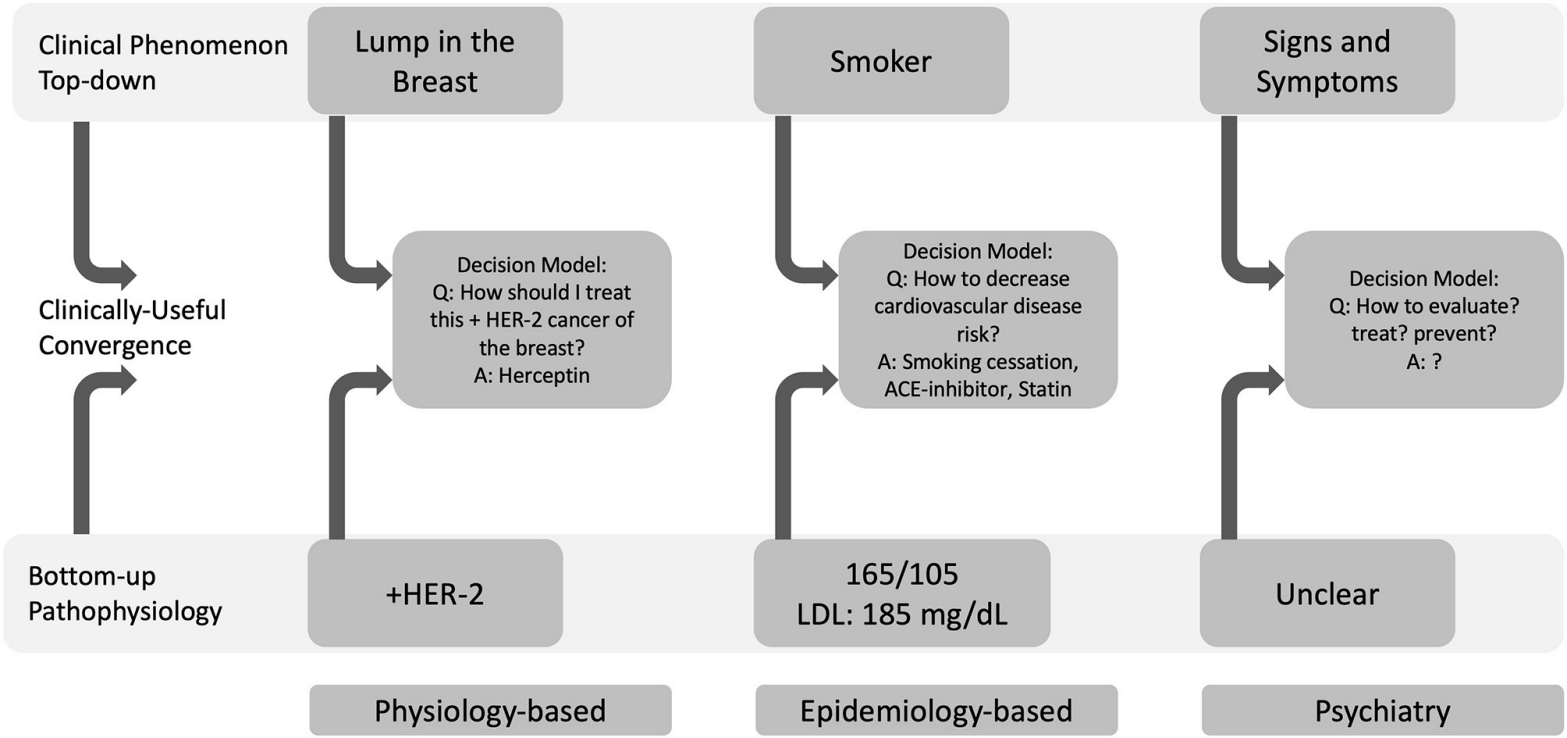
Physiology-based (bottom-up) and epidemiology-based (top-down) approaches to biomarker development can define useful decision models. Decision models converge top-down clinical phenomena with bottom-up pathophysiology. In oncology, a physiology-based investigation indicated that HER-2 positivity in a cancer of the breast could be treated with Herceptin. In cardiology, the Framingham Heart Study's epidemiology-based approach showed that smoking behavior (a top-down phenomenon), hypertension and high cholesterol were risk factors for cardiovascular disease. In psychiatry, it remains unclear how to define and operationalize decision models to approach clinical phenomena with pathophysiology and therefore, which data will be most helpful within these larger decision models (LDL, low-density lipoprotein).

The paragon of physiology-based biomarkers is the discovery of molecular disease markers in oncology. For centuries, cancer diagnosis and treatment were based on a decision model that was heavily weighted by where in the body the cancer was located. A patient might arrive in clinic describing symptoms of itching and tenderness over their breast. On their physical exam, a clinician might then note redness and lumps within the breast tissue, observable signs of advanced cancer. Cancer within the breast tissue was called “breast cancer” and was treated differently from cancer found elsewhere in the body. This decision model appeared straightforward, but treating cancer was capricious: two patients with breast cancer might have very different responses to the same treatment. The advent of tools to identify cell type and, subsequently, to create molecular tumor profiles that could probe the pathophysiology of cancer led to the discovery of the BRCA-2 and HER-2 gene mutations, which in turn showed that “breast cancer” was in fact a heterogenous mosaic of tumors (41). Moreover, molecular assays showed that mutations seen in some types of breast cancers were found in ovarian and prostate cancers. Such evidence demonstrated that a tumor's molecular profile could guide treatment selection. Today, cancers of the breast are routinely assayed for the HER2 molecular marker, which is directly associated with responsivity to Herceptin chemotherapy (42). Thus, HER2 is a biomarker that, in combination with other data guides a highly defined decision model toward effective treatment, as illustrated in Figure 2.

Exemplars of epidemiology-based biomarkers are blood pressure and blood lipid level. In combination with smoking, blood pressure and blood lipid levels are surrogate and modifiable risk factors of cardiovascular disease (CVD) (43, 44). President Franklin Delano Roosevelt's death from CVD led to the organization of the Framingham Heart Study in 1948 (44). At the time, little was known of CVD. Because little was known, it was unclear which data might be helpful in diagnosing, staging, or treating CVD; in other words, it was not clear how to define a useful decision model in CVD. Clear-cut clinical outcomes like a myocardial infarction were deemed invariably fatal and, without instruments to detect them, were diagnosed generally on autopsy (44). At that time, emerging research suggested the utility of electrocardiograms for diagnosing a myocardial infarction and for measuring blood lipids to predict MI risk. It was quite unclear which blood pressure was considered “normal” [at the time, the standard for normal systolic blood pressure was one's age plus 100 (43)]. Notwithstanding these knowledge gaps, the Framingham Heart Study's designers investigated all these seemingly disparate threads of evidence. In fact, they identified eighty phenotypic traits and measured them in 5,200 people. Over time, the Framingham Heart Study observed that cholesterol level, blood pressure, and smoking status formed a decision model that was associated with CVD at the population level (44). On the individual level, combining cholesterol level, blood pressure, and smoking status into a mathematical model of CVD led to the Framingham Risk Score (45), which described someone's risk of CVD given the magnitude of each measure (see Figure 2). The Framingham Risk Score subsequently generalized to novel populations (46, 47). Even though the decision model was identified at the population-level, the Framingham Risk Score is widely used by clinicians to guide treatment for individual patients and has guided decades of drug development (45) (https://www.mdcalc.com/ascvd-atherosclerotic-cardiovascular-disease-2013-risk-calculator-aha-acc). Blood pressure and blood lipid levels are therefore biomarkers that, in combination with smoking status, form a decision model that guides clinical action by suggesting lifestyle modifications and pharmacologic treatments.

And yet, the presence of a cancer or a myocardial infarction is a binary distinction for disease: you either have it or you do not. But the line between health and disease is not always obvious, particularly for disorders of emotion, thought, and behavior (4). In psychiatry, the assumption that health and disease are discrete categories is being replaced by the observation that phenotypes present across a population are shifted toward extremes in disease (48). Similarly, observations that individual phenotypes can vary greatly across a population have led to the view that there is no universally optimal (or “healthy”) profile of brain function (4). Though a distribution of continuous phenotypes might erode confidence in the possibility of categorical diagnoses in psychiatry, this has not been the case in other specialties: hypertension is parameterized as a range of blood pressures. Continuous phenotypes have the added value of expressing magnitude, which could be especially relevant given the multifactorial nature of psychiatric disease and the possibility that a decision model might draw probabilistically on multiple forms of (continuously measured) biomarkers.

Weakly specific and weakly sensitive biomarkers can guide clinical action if the decision model and, crucially, the decision in question is sufficiently well-defined. HER-2 gene positivity indicates that the drug Herceptin may be helpful in treating a very specific form of cancer. HER-2 positivity, therefore, resolves a specific treatment decision in a specific decision model: HER-2 does not resolve the treatment decisions in a “hot and shaky” patient or in a torn anterior cruciate ligament, or even in the selection of alternatives to Herceptin in a HER-2 positive breast cancer. Similarly, identifying risk factors for CVD guides clinicians to measure, trace, and then target those factors with treatment. HER-2 and CVD risk factors therefore are biomarkers that inform specific, well-defined treatment decisions within larger, well-defined decision models.

Framing clinical decision from a Bayesian perspective illustrates that, to be “fit for purpose,” a biomarker must operate within a well-defined decision model to: (1) provide the clinician data they cannot currently access; (2) guide the collection of additional data; (3) uniquely resolve a well-defined treatment decision; (4) provide a convergence between top-down clinical phenomenology and bottom-up pathophysiology. Accordingly, we argue that operationalizing decision models in psychiatry is crucial if researchers hope to offer a biomarker to inform optimal decision-making (49).

## Operationalized Decision Models

Psychiatry lacks operationalized decision models. This does not mean that psychiatry *has no* decision models. We reason that individual clinicians successfully treat individual patients by forming their own decision models that then guide data collection and treatment selection. In other words, psychiatrists treat patients by acting on idiosyncratic decision models. What psychiatry lacks is a way to formally describe an idiosyncratic decision model, thereby allowing it to be shared, evaluated, and optimized in terms of efficacy and efficiency. In machine learning and cognitive science, the process of optimizing a decision or generative model^1^ is known as *structure learning* or—in statistics —(Bayesian) *model selection* and is one of the most important and difficult problems in the field (50–55).

Consider what happens when a clinician receives this one-line report on an intake form: “a 50-year-old man with schizophrenia is speaking to his dead girlfriend.” This sentence serves as the first piece of information that, based on her clinical training, forms her preliminary decision model. If the clinician knows only that a man is speaking to his dead girlfriend, her preliminary decision model might include several hypotheses: e.g., normal or pathologic grieving, intoxication, withdrawal, trauma, or some other “organic” brain disease perhaps even a bacterial meningitis. Knowing that the patient is a 50-year-old man with schizophrenia makes a primary psychosis more likely and so what is required from a Bayesian perspective is data to eliminate the less likely but more serious hypotheses that require immediate intervention (e.g., delirium tremens from withdrawal) and to confirm a more likely hypothesis (schizophrenia). A series of standardized laboratory tests—a urine toxicology, breathalyzer, complete blood count, or blood electrolytes—would help rule out the less likely albeit more serious and easily treatable disease hypotheses. The clinician values these tests because the reliability (sensitivity, specificity, accuracy, range of error, and uncertainty) of the assays upon which they are based are regularly monitored and calibrated.

From a Bayesian perspective this is the problem of optimum Bayesian design (12, 16) or, active (Bayesian) inference (56, 57): the clinician uses standardized laboratory tests to eliminate less likely hypotheses to render the “true” disease hypothesis—and therefore the treatment decision—more certain or precise. And yet, if the clinician wanted to increase her confidence that the 50-year-old man indeed has schizophrenia, there are no standardized clinical tests she could perform. The clinician would simply ask her patient whether he experienced specific negative or positive symptoms of schizophrenia and for how long. During this conversation, the clinician would carefully observe the patient's demeanor, dress, affect, behavior, and thought process, looking for signs of schizophrenia such as blunted affect, disheveled appearance, and disorganized thought process. As the clinician accumulates more data, her relative certainty of a primary psychosis might increase—and her uncertainty about how to treat the patient would resolve. In sum, her decision model helps her organize and seek out new data, guiding her to a decision.

Framing clinical decisions with Bayesian inference allows the decision model itself to be made explicit and optimized. The clinician's decision model and implicit prior beliefs can, in principle, be operationalized by mapping backwards from her final decision to the data that preceded it. In other words, it is possible to make an *objective* inference about the clinician's *subjective* inference by defining which decision model would make her ultimate decisions the most likely. This approach has already been established at the level of proof of principle in computational psychiatry, where the focus is to infer the prior beliefs of experimental subjects and, ultimately, patients using their behavioral responses to various stimuli and economic games (15, 19). However, the same procedures can, in principle, be applied to the psychiatrists using their diagnostic and treatment responses. Note the subtlety of this approach; namely, treating psychiatrists as expert Bayesian inference machines and reverse engineering the decision models that underwrite their diagnostic skills. The idea here is to operationalize decision models by making them explicit—by identifying the decision model that best explains the diagnostic behavior of one psychiatrist or another. As sentient creatures, with theory of mind, we do this all the time: for example, one can often infer what another person is thinking by watching where they are looking in a particular context. The notion here is that it could be mathematically applied to the diagnostic behavior of psychiatrists.

Operationalized decision models allow performance to be measured within and across individuals. Measuring how one clinician operates over time might identify decision efficacy and efficiency that, as expected by behavioral economists, varies with the time of day, the clinician's mood or whether they've eaten lunch or had their coffee (58). Model comparison further allows two clinicians' decision models to be formally compared by how efficiently they guide data discovery and by how effectively they arrive at a treatment decision which benefits the patient, which is further measured by clinical data.

In the case of our 50-year-old man, we could operationalize a decision model that excludes intoxication, withdrawal, or other “organic” brain diseases. This process can be operationalized because each value within a laboratory test is quantified and can therefore be mathematically modeled. We could not, however, operationalize a decision model that confirms a primary psychosis because the symptoms and signs of schizophrenia upon which a diagnosis of schizophrenia is based are not quantified (see Table 1). Because the symptoms and signs are not quantified, the accuracy, range of error, and uncertainty of any specific datapoint cannot be ascertained, further complicating their inclusion in an operationalized decision model.

Symptoms cannot be quantified because, by definition, they are the patient's personal experience that, in turn, relies on the patient's cognitive ability to sense, interpret, and report that experience. The reliance on self-report assumes that the relevant drivers of behavior are accessible linguistically to the reporter. But people are unaware of many of the drivers of their behavior (something called anosognosia) and, further, some forms of behavior (e.g., habits) are not represented by linguistic circuits in the way that goal-directed behaviors are and, therefore, remain difficult or impossible to articulate (59, 60). Although some symptoms are only detected by a patient's report (e.g., hallucinations), a reliance on self-report is problematic: in psychiatry, we often rely on a patient's perception of reality to diagnose disorders of reality perception.

Observable signs also rely on clinical inference. Two clinicians can observe the same patient and might disagree about whether the patient's thought process was “disorganized.” And even if two clinicians agree that the patient's thoughts are “disorganized,” there is no measure for *how disorganized*. Furthermore, because there is no empirical way to demonstrate how each clinician's brain *detected* the disorganization in the patient's speech (i.e., which specific words, phrases, or string of ideas in the patient's speech led each clinician to conclude the speech was disorganized), it is difficult to determine whether two clinicians agree on what “disorganized” means or whether two clinicians believe the patient's speech was disorganized for the same reason. Essentially, because we do not have direct access to the raw data a clinician solicits during her clinical exam, we cannot use model inversion to identify her data discovery procedure. An unfortunate corollary of this problem is that, right now, if a clinician attempts to treat a patient's disorganized speech with, say, an antipsychotic, there is no way to objectively ascertain whether and how much the disorganization changes with that treatment.

In sum, in the absence of quantified clinical data, we cannot operationalize how a clinician arrives at a treatment decision or how to modify treatment as the decision model updates. And in the absence of a clearly defined decision model, it is quite unclear where a biomarker might be of use. This means that the prerequisite to defining a biomarker to formalize decision models is to first develop quantitative phenotypes. We describe how this might proceed in two stages, below.

## Practical Stages of Phenotyping and Decision Modeling

Before a biomarker can inform clinical decision, that clinical decision process must itself be explicitly formalized and evaluated. Put differently, we argue that a precondition to biomarker development is that clinically salient data be rigorously quantified and that clinical decisions be operationalized and evaluated based on those data (61). Only when both preconditions are met can statistical analyses be performed to determine *which* data are the most useful for *which* decisions. And yet it remains unclear for psychiatry which data might be the most useful to acquire and analyze.

Psychiatry is in a conundrum comparable to where the designers of the Framingham Heart study found themselves in the late 1940's: we have multiple disparate lines of thinking about the causes of mental illness that are now only beginning to coalesce into tenable hypotheses (62). Promising analyses of even the largest samples with supposedly promising statistical power and high statistical significance have repeatedly failed on the individual level (26, 63, 64). Though this failure is often attributed to the high phenotypic variability of psychiatric patients, it is worth noting again that clinicians nevertheless successfully recognize salient data and treat psychiatric illnesses on the individual level by applying their own idiosyncratic decision models.

Broadly speaking, as a field, we feel confident that facets of a patient's biological, social, and psychological history are relevant to the behavioral expressions of mental illness that we treat (40). Yet behavior itself remains a vague and poorly defined phenomenon. Behavior—whether reported by patients or observed by clinicians—is not objectively measured in a way comparable to the molecular assays, blood tests, or electrocardiograms prevalent in other medical specialties. Here, psychiatry might benefit from digital technologies that have recently emerged specifically to quantify human behavior. We reason that digital tools might help psychiatry in two stages: stage 1 would quantify data already present in the standard clinical interaction and allow decision models to be operationalized and evaluated; stage 2 would explore whether other forms of data not currently used in the clinical evaluation might have value within an operationalized decision model.

Stage 1 would quantify clinical data and operationalize the decision models currently employed in clinical practice (see Table 2). Before moving ahead to define new forms of data or combinations of data that *might* be relevant to clinical work (as described in stage 2), the field should instead quantify those behaviors and operationalize those decisions that we already agree are clinically relevant as rigorously as possible.

**Table 2:** Digital phenotypes are quantitative observable signs.

**Qualitative data**		**Quantitative data**

**Subjective symptoms**	**Observable signs**	
**Patient report**	**Clinician observation**	**Digital phenotypes**
What's on your mind?		Search history, social media
What's your typical day like? How active are you? How much sleep do you usually get?		Actimetry, geolocation
Are you a social person? How are your relationships?		Call/text logs, social media profile
How's your mood throughout the day?		
	Affect, appearance, attitude	Facial action unit motion and fluidity analysis
	Affect, speech, thought content, thought process	Semantic analysis, natural language processing, vocal acoustics
	Psychomotor behavior	Head box analysis

*This table illustrates how commonly assessed qualitative data like subjective symptoms reported by patients and observable signs observed by clinicians can be quantified as digital phenotypes. Quantitative data has the added value of being able to operate within Bayesian decision models*.

Stage 1 would involve creating video and audio recordings of clinical interactions and using digital tools to measure the data a clinician already solicits during her exam (65). A video recording can be separated into visual data and audio data. Visual data can be processed to label different parts of the body, allowing the speed, acceleration, fluidity, and coherence of movement to be measured. Facial expression can be quantified by measuring how different facial action units coordinate over time (66); not necessarily to label emotional state, but rather to measure how an individual's unique repertoire of facial expression changes over time. Body language—both the patient's and the clinician's—can be quantified as the relationship of the head to the shoulders, torso, and legs throughout the clinical interview (67). Voice data can be analyzed for its acoustic properties to measure how often a patient takes a breath, how many syllables they utter per second, how their intonation changes over time (68). Speech can be transcribed and measured using tools that can define semantic and psycholinguistic content (69–71). In essence, the mental status exam can, in theory, be measured with digital tools (66).

Practically, if a clinician was evaluating a “50-year-old man with schizophrenia is speaking to his dead girlfriend,” she would proceed with her exam as usual except a video would record her interaction. Such a recording would capture the same data she is sensing with her eyes and ears except it will now be digitally. A host of mathematical tools can be applied to this digital data. In addition, the clinician's decision model can be inferred and formalized using Bayesian methods described above. Crucially, different decision models—whether from two different clinicians or from the same clinician at different timepoints—can be compared and optimized using the same Bayesian methods, thereby leading to decision models that are more efficient and effective. Once the data and decision models clinicians currently use have been formally evaluated, new forms of data can be evaluated.

Stage 2 would evaluate whether forms of data not currently used in clinical decision might add value at specified points in larger decision models. As outlined in Table 1, relatively new technologies such as aggregates of someone's online search (72) or social media history (73–77) might inform clinicians about how a patient's interests, self-esteem, or social relationships change over time. Paired with geolocation data, actimetry tools offer measurements for how active a patient is (78, 79), how much they are sleeping, and how these both change over time (80, 81). Daily call and text logs can provide a measure of a patient's social connectedness and engagement (82, 83). Furthermore, wearable sensors that detect heartrate variability and skin conductance during or between clinical encounters could provide a measure of a patient's stress response and how this response changes with treatment (84).

In addition, other forms of data that are not currently used in clinical practice—such as genetic information or exposome—could be more ably evaluated within an operationalized decision model. Even though each datapoint might have limited specificity and sensitivity in isolation, in combination, they might have utility at a specific decision within a larger decision model.

Rigorous measurements of behavior in the clinical setting—and especially outside of it—can help psychiatrists obtain more naturalistic and nuanced data, yet we acknowledge that it is not clear which aspects of behavior, at what time frequency, or for how long such data should be collected (85, 86). This is analogous to the measurement of body temperature of our “hot and shaky” patient treated with antibiotics; we would expect the temperature to vary over the course of the treatment. However, unless temperature is ascertained sufficiently frequently, there is no way of objectively knowing the time scale of the infection. Determining a suitable time scale (days, months, years, or decades) is largely dependent on having access to an operationalized decision model within which time scale has utility. Sampling a patient's temperature over the course of a day or week might inform a specific decision like antibiotic selection (e.g., if a patient remains febrile, it is likely an antibiotic is ineffective against a given infection), but measuring temperature over the course of a month would not.

## Phenotyping, a Prerequisite to Biomarker Development

The relatively nascent field of Deep Phenotyping aims to collect data for large, longitudinal samples using standardized and rigorous procedures (87). Multiple on-going, large-scale, necessarily collaborative efforts are seeking to provide deep phenotypes (88) that span genetic and epigenetic data to brain imaging to digitized behavioral and online data (89, 90). Together these data seek to measure—as much as possible—an individual's biologic, social, and psychologic profile.

Although it is unclear which types of data will prove the most relevant, it is clear what we need to learn from these data: how to quantify, monitor, and modify specific decision models. The first step to modifying the course of any illness is to fully characterize that illness as it develops from health, similar to what the Framingham Heart Study has done for cardiovascular illnesses (44, 61). It is worth noting that the Framingham Heart Study did not discover physiologic concepts like cholesterol or blood pressure; these were known prior to the initiation of the study. Rather, the Framingham Heart Study motivated further investigation of this physiology by connecting it to clinical phenomenology, thus bridging bottom-up and top-down clinical evaluation. Put differently, the identification and appreciation of precise physiologic mechanisms underlying cardiovascular illnesses came only after a precise clinical decision model was defined and traced over time.

Likewise, the identification of the HER-2 biomarker required a decision model based on clinical interview (subjective symptoms), routine physical exam (observable signs), mammography, and biopsy. Only with a carefully defined phenotype was HER-2 able to add value to clinical decision by converging bottom-up and top-down assessment within a single, highly specific treatment decision: whether to prescribe Herceptin.

Therefore, as psychiatry's bottom-up understanding of pathophysiology continues to evolve, our top-down measurement and formalization of clinical phenomenology will become ever more crucial if the two fields of inquiry are to converge in meaningful ways. Though it is possible that the individual genes or pathophysiological pathways underlying clinical conditions will be associated with specific diagnoses or subtypes, this seems unlikely to be broadly applicable—given the diagnostic ambiguity across psychiatric diagnoses, the multi-determined nature, and the unclear decisions a candidate biomarker would address (91, 92). Operationalized decision models informed by quantitative phenotypes appear to be a way forward.

Endeavors of this scope and magnitude require significant investments of time and resources. Yet it is worth bearing in mind that the amount of time and resources required to gather background information necessary to provide actionable insights are an investment for future generations. Defining clinical decision models to guide treatment in the presence of disease have the added benefit of informing decision models to guide prevention before that disease emerges.

For patients, history has shown that early diagnosis and preventative treatment can alter certain disease trajectories, thereby creating an enormous benefit across a population that more than justifies the costly upfront investment (44). It is true that the development of new technologies can increase the proximal cost of healthcare delivery. At present, because the vast majority of generic psychiatric medications are inexpensive and offer palliative treatment to a broad category of patients, it may be more economic and effective to broadly offer palliative treatments than to deeply phenotype patients in an effort to identify modifying or curative treatments that help only a relative few (see Table 1). However, overall healthcare costs can be decreased by more effectively identifying and treating illness in the preventative stage (93). Overall, new technology (once effective) can demonstrate which preventative health measures might best promote health or reduces the economic burden of illness by decreasing inpatient admission and increasing public health and productivity. An additional added benefit of technology is that its cost decreases over time, thus expanding access to populations who previously had been unable to benefit from the healthcare for reasons of cost, geographic, or equity.

For researchers, laying a rigorous foundation for data collection, synthesis, and modeling will produce a dataset that can inform a multitude of studies, which (if the Framingham Heart Study is any indication) can yield large and compounding dividends for the scientific community. Although it is not clear what time scale and data will prove to be the most beneficial for psychiatry, what makes such an investment timely for behavioral and mental health is the fact that the necessary tools and techniques for such a study have only recently emerged.

In summary, psychiatry has yet to develop and validate biomarkers that improve clinical practice. This report represents an attempt to step back and consider why our past efforts to develop biomarkers have failed and to reframe our efforts in terms of data and decision science. As our bottom-up understanding of the pathophysiology of psychiatric illnesses continues to evolve, our top-down measurement and formalization of clinical phenomenology will become ever more crucial if the two fields of inquiry are to converge in meaningful ways. Step toward this convergence include first, rigorously quantifying clinical data and operationalizing existent psychiatric decision models and, second, evaluating where new forms of data, including candidate biomarkers, might be of value. Our hope is that making clinical decision explicit will reframe the biomarking enterprise so it might impact clinical inference and, in turn, improve the lives of our patients.

## Author Contributions

DSB and JK conceptualized and drafted the manuscript. JB, KB, DB, SE, KF, PF, PG, SH, AH, J-PO, AP, and DS edited and provided input. All authors contributed to the article and approved the submitted version.

## Funding

DSB was partially funded by the National Institute of Mental Health (5 T32 MH 19961-22).

## Conflict of Interest

SH was employed by company T. J. Watson IBM Research Laboratory. The remaining authors declare that the research was conducted in the absence of any commercial or financial relationships that could be construed as a potential conflict of interest.

## Publisher's Note

All claims expressed in this article are solely those of the authors and do not necessarily represent those of their affiliated organizations, or those of the publisher, the editors and the reviewers. Any product that may be evaluated in this article, or claim that may be made by its manufacturer, is not guaranteed or endorsed by the publisher.

## Footnotes

^1^Namely, a model that generates consequences from causes; in our setting, a model that generates signs, symptoms, and biomarkers from the right kind of psychiatric nosology. Inverting a generative model is the same as inferring the diagnosis, given the observable or measurable consequences of a psychiatric condition.
